# Evaluation of Performance and Stability of a Gel-Type Polymer Sorbent for Recovery of Phosphate from Waste Streams

**DOI:** 10.1021/acsapm.4c03237

**Published:** 2024-12-06

**Authors:** Michela Pacchione, Lucas Urbano José, Ulla Gro Nielsen, John W. McGrath, Panagiotis Manesiotis

**Affiliations:** † School of Chemistry and Chemical Engineering, Queen’s University, David Keir Building, Stranmillis Road, BT9 5AG Belfast, Northern Ireland, U.K.; ‡ Department of Physics, Chemistry and Pharmacy, University of Southern Denmark, Campusvej 55, 5230 Odense, Denmark; § School of Biological Sciences, Queen’s University, 19 Chlorine Gardens, BT9 5DL Belfast, Northern Ireland, U.K.

**Keywords:** polymer sorbents, anion exchange, solid-state NMR, phosphate, nutrient sustainability

## Abstract

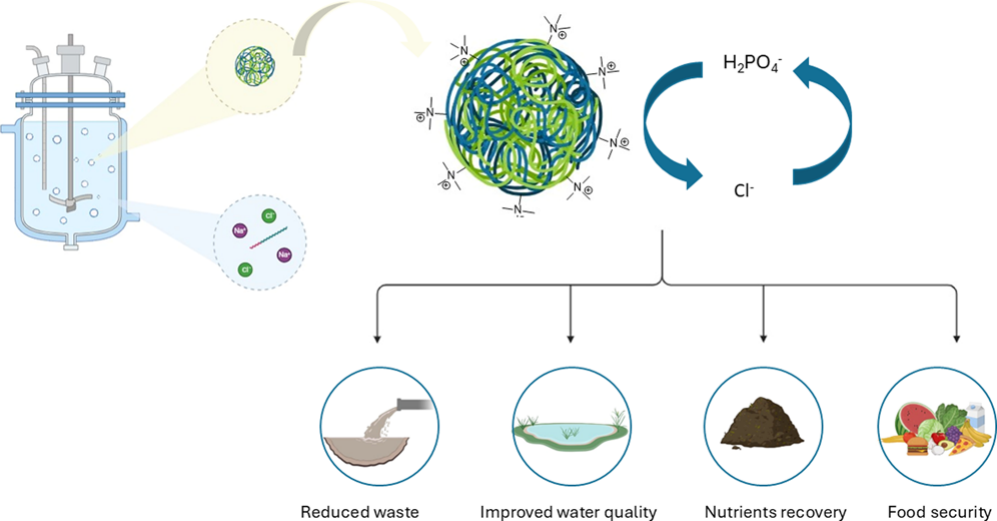

Phosphorus (P) fertilizer is an essential component of our food system with the majority of all mined P rock processed to make mineral fertilizers. Globally however P rock stocks are declining—both in quality and quantity—with poor P management creating a linear economic system where P is mined, globally redistributed into products and eventually discharged into the environment leading to eutrophication. To enable establishment of a circular P economy, whereby P can be recovered from waste for its industrial reuse, requires the development of effective P recovery technologies. Adsorption via anion exchange is a promising technology for P recovery from waste streams; however, in many cases, the regeneration of the sorbent used (to allow for its subsequent reuse) has not been extensively investigated. We now describe a gel-type anion exchange polymer composed of vinyl benzyl chloride, hydroxypropyl methacrylate, and ethylene glycol dimethacrylate synthesized via suspension polymerization. The obtained material was modified to introduce a quaternary ammonium functionality, characterized, and tested for phosphate capture, in the presence of competing anions and from wastewater samples. Fixed-bed experiments were conducted using monobasic phosphate as adsorbate to test polymer performance, and its stability upon repeated adsorption/desorption cycles was investigated by solid-state NMR, revealing that no degradation or loss of P binding performance across 50 regeneration cycles. Excellent polymer performance in the recovery of P from wastewater was also demonstrated.

## 1 Introduction

Phosphorus (P) is a crucial element in food production, mainly used for the formulation of fertilizers in its orthophosphate form.^1^ Fertilizer production relies on the mining of phosphate rock, which is a finite natural resource. Globally both the quantity and quality of P rock stocks are declining while the mining process itself is environmentally challenging. This is due to the high amount of waste clay produced, which contains high concentrations of nutrients (primarily P) and heavy metals.^2−4^ Poor phosphate management is also an issue concomitant to the increased use of phosphate fertilizers across the agri-food sector. Intensive agriculture and the overuse of synthetic fertilizers has resulted in diffuse phosphate pollution, which results in the eutrophication of freshwater systems.^5,6^ Such environmental phosphate pollution is further exacerbated by the release of untreated or poorly treated wastewater into water bodies.^7^ Indeed anthropogenic eutrophication poses the most significant challenge to water quality globally, threatening ecosystems, biodiversity, and human health.^8−10^

Recycling of P from waste plays a key dual role in the development of sustainable P management strategies, to ensure both the maintenance of its supply for global food production and in the prevention of P pollution. Current P recovery technologies are primarily applied in wastewater treatment plants, enabling P recovery from the aqueous phase, generally through precipitation as struvite. P recovery from sewage sludge can also be accomplished via crystallization to produce vivianite, or through processes that involve dewatering and incinerating the sludge, followed by phosphorus leaching using acids.^11^ The recovered P is then converted into products like phosphoric acid or struvite.^12,13^

In addition to precipitation and crystallization approaches adsorption-based methodologies have a strong potential for P recovery due to their ease of operation, scalability, lower energy requirements (when compared with techniques which require an incineration step) and versatility, as they can be implemented at various stages of wastewater treatment. Moreover, these technologies can remove P to very low concentrations within aqueous solutions (10–100 μg L^–1^). Such low P concentrations are difficult to achieve using precipitation methods.^14,15^

To date most phosphate sorbents on the market contain iron, which binds phosphate by inner-sphere complexation. These sorbents mainly take the form of iron oxides or hybrid anion exchange resins, both of which require extensive washing with caustic solutions to be regenerated.^16^ Few adsorption polymer studies have however fully investigated these polymer regeneration steps and for those that do the studies are limited to a few cycles. Kumar et al. have previously shown that for an absorption polymer to be economically valuable for P recovery, the adsorbent should be reused at least 50–100 times.^15^ Moreover, those studies reporting the regeneration of adsorption polymers only consider the variation in binding capacity or selectivity; the chemical structure and degradation mechanisms that might be involved have not been investigated.^17,18^

Solid-state NMR (ssNMR) is a powerful characterization technique that has been applied to the analysis of a vast range of compounds and materials in their native state, i.e. without the need to dissolve or otherwise treat the sample. However, its application in polymer chemistry, especially anion exchange materials, is not widely explored. To date, no reports on polymer sorbent regeneration have used ssNMR to investigate whether changes in polymer structure or chemical composition (and thus binding capacity) occur as a consequence of the regeneration protocol employed^19−21^ (although some studies have focused on monitoring polymer hydrolysis and degradability in the dry or hydrated state). In the latter context it is generally ^1^H single pulse (SP) and ^13^C cross-polarization (CP) magic angle spinning (MAS) experiments which are the main characterization tools used.^22,23^ Nevertheless, most studies use ssNMR only to characterize the material without investigating the reusability of the resin.^24^

Spin–lattice relaxation in NMR refers to the process by which an excited spin returns to equilibrium, and for homogeneous solid samples, this process can be described by the spin–lattice relaxation time, T_1_.^25^ T_1_ can give information on the degree of mobility of polymers, and it is frequently used to monitor the degradation of a polymer, as a function of temperature or of a degrading agent, such as a strong acid or base.^26,27^ The established method reported in the literature to measure T_1_ is through inversion–recovery experiments, however, most studies are limited to measuring the ^1^H T_1_, as acquiring ^13^C T_1_ through direct excitation is time-consuming—due to the low natural abundance of ^13^C.^28^ Nevertheless, the measurement of the ^13^C relaxation time via ^1^H through cross-polarization can still give valuable information about the mobility of the ^13^C nuclei.^29^ Along with a material’s regenerability, testing in the presence of competing anions and real samples can provide valuable information regarding the competitively of organic or inorganic compounds for binding. This has been extensively reported for hybrid anion exchange resins.^30,31^

In the present study, a gel-type polymeric material for phosphate capture was developed and synthesized via suspension polymerization. This was then reacted with a tertiary amine (trimethylamine) to introduce an anion-exchanger quaternary ammonium functional group. The functional monomers, hydroxypropyl methacrylate (HPMA) and vinylbenzyl chloride (VBC), were selected to introduce a neutral hydrophilic moiety that prevents charge repulsion and increases the hydrophilicity of the material, and a readily functionalizable group (R–CH_2_–Cl) into the polymer structure, respectively. The cross-linker, ethylene glycol dimethacrylate (EGDMA), was chosen to enhance hydrophilicity compared to commercially available resins. Finally, functionalization with trimethylamine (TMA) introduced a quaternary ammonium group, a common feature in anion exchange materials, facilitating comparisons between the synthesized polymer and those reported in literature. The resulting material (VBC–HPMA–EGDMA–TMA) was tested for phosphate binding. The polymer was used in a fixed bed process to assess phosphate removal from a model phosphate solution and was regenerated 50 times to evaluate how many regenerative cycles it could undergo while still retaining its performance. A comprehensive structural analysis using ssNMR was also performed, to investigate any changes in the polymer structure after repeated use and regeneration. ^31^P SP MAS spectra were acquired to confirm the complete phosphate desorption, while ^13^C CP MAS was used to verify the retention of the chemical structure after regeneration. ^1^H T_1_ and ^13^C CP T_1_ were acquired to provide insights into the polymer matrix. Monitoring the T_1_ at the different regeneration cycles gave a unique insight into the nuclei mobility change with the regeneration cycles. To our knowledge, this is the first comprehensive study of the performance and stability of a phosphate-binding polymer using ssNMR. Finally the material’s performance was assessed both in the presence of competing anions, and in the recycling of P from domestic wastewater.

## 2 Experimental Section

### 2.1 Materials and Monomers Purification

All chemicals used, including the multianion standard for IC, were purchased from Sigma-Aldrich (Gillingham, UK). To remove any potential polymerization inhibitors, the 4-VBC, ethylene glycol dimethacrylate (EGDMA), and hydroxypropyl methacrylate (HPMA) were treated via gravity filtration through a 25 mL syringe packed with basic alumina. 2,2′-Azobis(2-methylpropionitrile) (AIBN) was used as received.

### 2.2 Polymer Synthesis

The polymer was synthesized by suspension polymerization in a 2 L jacketed reactor, equipped with a circulating bath and overhead stirrer. The aqueous phase consisted of a 1.06% (w/w) poly(vinyl alcohol) (PVA) solution, prepared by initially dissolving 9 g of PVA in 837 mL of ultrapure water and heating at 90 °C overnight. The solution was transferred to the reaction vessel, where the temperature was adjusted to 70 °C and the stirring speed set to 250 rpm, followed by the addition of 54 g of NaCl to yield a 1% (w/w) PVA/6% NaCl (w/w) solution. The oil phase was prepared by mixing 30.8 mL of purified VBC, 4.6 mL of purified EGDMA, and 2.6 mL of purified HPMA in 60 mL of toluene. 0.28 g of initiator AIBN was added, and the prepolymerization mix was sonicated for 15 min to remove dissolved oxygen. Subsequently the oil phase was poured into the preheated aqueous phase, and the reaction was allowed to proceed under stirring for 8 h. The polymer beads formed were collected, washed with hot water and sieved, to separate the different size fractions.

### 2.3 Functionalization with Trimethylamine

Five g of the obtained beads (120–250 μm) were transferred into a round-bottom flask, equipped with a magnetic stirrer. The beads were stirred in ethanol at 200 rpm and 25 °C for 1 h. After this time, 10 mL (1.5 equiv) of 4.2 M trimethylamine were added to the suspension. The suspension was heated at 60 °C and stirred for 24 h. After this time, the beads were filtered to remove the excess solution and dried in an oven at 60 °C for 4 h and in a vacuum oven at 50 °C overnight. A comprehensive reaction scheme for the polymeric beads synthesis is described in the Supporting Information (Figure S1: Synthesis scheme for the polymeric beads).

### 2.4 Characterization

FTIR spectra were acquired using an Agilent Cary 630 FT-IR spectrometer at a wavelength of 15,385–2500 nm and 64 scans, and processed using spectrograph. Elemental analyses (CNHS) were performed using a PerkinElmer PE2400 CHNS instrument, with combustion temperature set at 975°C. Scanning electron microscopy (SEM) images were acquired using a FEI Quanta FEG—Environmental SEM. Before the measurement, the samples were gold-coated under vacuum for 80 s and measurements were taken at 10 and 20 kV.

### 2.5 Ion Chromatography

All liquid samples were analyzed using a Thermo Dionex ICS-1000 ion chromatography (IC) system, equipped with an AS–AP autosampler, a heated conductivity cell, and an AERS 2 mm suppressor. The column used was a Dionex IonPac AS-22 fast, equipped with an AG-22 guard column. The eluent was 4.5 mM sodium carbonate/1.5 mM sodium bicarbonate, and a flow rate of 0.3 mL/min was used. The injection volume was 20 μL.

### 2.6 Binding and Kinetic Isotherms

Binding isotherm studies were conducted by placing 0.06 g of polymeric material into 20 mL scintillation vials. Then, 10 mL of a KH_2_PO_4_ solution with concentrations of 25, 50, 100, 150, 300, 600, 1000, or 1500 mg L^–1^ were added to the vials. The vials were closed and agitated gently at 25 °C for 2 h. After this time, 1.5 mL of solution was withdrawn, filtered using 0.22 μm membrane filters, and analyzed by IC. Binding isotherms (*n* = 3) were constructed by plotting the amount of bound phosphate vs the amount of free phosphate in each supernatant, and data points were fitted to the langmuir equation.

Binding kinetics were investigated by placing 0.06 g of material into 20 mL scintillation vials and adding 10 mL of a 300 mg L^–1^ KH_2_PO_4_ solution. The vials were closed and placed into a roller agitator at 25 °C. 1.5 mL of solution was withdrawn at 5, 10, 15, 20, 40, 60, 80, 100, 120 min, filtered through 0.22 μm membrane filters and analyzed using IC. All experiments were performed in triplicate. The data points obtained were fitted to the pseudo-first-order equation.

Competition of other anions for the binding was investigated by placing 0.06 g of material into 20 mL scintillation vials and adding 10 mL of a 300 mg L^–1^ KH_2_PO_4_ solution and different concentrations of the competing anion (50, 150, and 300 mg L^–1^). The anions studied were chloride (Cl^–^), nitrate (NO^3–^) and sulfate (SO_4_^2–^). The vials were closed and placed into a roller agitator at 25 °C. After 2 h, 1.5 mL of solution was filtered through 0.22 μm membrane filters and analyzed using IC. All experiments were performed in triplicate.

### 2.7 Flow Experiments

0.5 g of polymer beads were packed in a 12 mL SPE cartridge. A 100 mg L^–1^ H_2_PO_4_^–^ solution was passed through the cartridge using a 1.8 mm connection tube, with a flow rate of 6.7 mL min^–1^. The phosphate solution was passed through the SPE column for 120 min and effluent fractions (1 mL each) were collected at established times (5, 10, 15, 20, 30, 40, 50, 60, 70, 80, 90, 100, 110, 120 min) and analyzed using IC. The regenerant solution used was 0.1 M HCl, which was passed through the column at 6.7 mL min^–1^ for 50 min. The experiment was repeated after different regeneration counts (1 cycle of regeneration, 3, 5, 10, 20, 30, and 50 cycles), to collect the beads for analysis. The beads were dried in the vacuum oven at 50 °C overnight and used for the NMR experiments. Phosphate removal (*n* = 3), expressed as %, was calculated at the end of each cycle, as , where n is the number of sampling points per cycle (*n* = 14), C_0_ is the initial H_2_PO_4_^–^ concentration (100 mg L^–1^) and *C*_t_ the concentration at the sampling time.

### 2.8 Solid-State NMR

Solid-state NMR spectra before and after polymer functionalization were recorded on a Bruker Avance 500 MHz (11.7 T) NMR spectrometer using a 4 mm triple resonance MAS NMR probe. ^1^H [*v*_0_ (^1^H) = 500.13 MHz] spectra were obtained via single pulse excitation (90° pulse, 2.5 μs), 3 s relaxation delay, 4 scans and 12 kHz spinning. Deionized water was used as a chemical shift reference [(δ_iso_^1^H) = 4.6 ppm]. All the ^13^C [*v*_0_ (^13^C) = 125.76 MHz] CP spectra were obtained via cross-polarization (2.78 μs pulse, 2 ms contact time, 4 s relaxation delay, 4096 scans and 12 kHz spinning). Adamantane was used as a chemical shift reference [δ_CH2_ (^13^C) = 38.5 ppm] and peaks were assigned based on the monomers’ corresponding solution state ^13^C NMR spectra.

Solid-state NMR spectra at the different regeneration cycles were recorded on a JEOL ECZ 500 MHz (11.7 T) NMR spectrometer using a 3.2 mm triple resonance MAS NMR probe. ^1^H [*v*_0_ (^1^H) = 500.13 MHz] spectra were obtained via single pulse excitation (90° pulse, 3.15 μs), 3 s relaxation delay, 4 scans and 15 kHz spinning. Deionized water was used as a chemical shift reference [δ_iso_ (^1^H) = 4.6 ppm]. ^31^P [*v*_0_ (^31^P) = 202.46 MHz] spectra were obtained via single pulse excitation (45° pulse, 3.1 μs), 10 s relaxation delay, 5500 scans and 15 kHz spinning. 85% phosphoric acid was used as a chemical shift reference [δ_iso_ (^31^P) = 0 ppm]. ^1^H spin–lattice relaxation times (T_1_) were measured using the inversion recovery pulse sequence (180_*x*_ – τ– 90_*x*_) and the FID was acquired after the 90° pulse. Four scans were acquired at each τ (48 points, so total scans 192), with 20 s relaxation delay and 15 kHz spinning. ^13^C CP T_1_ were obtained via inversion recovery (180_*x*_ – τ–90_*x*_) followed by cross-polarization (3.15 μs pulse, 2 ms contact time), with 10 s relaxation delay, 128 scans (48 points, so total scans 6144) and 15 kHz spinning. For both experiments, the curve obtained by plotting the time (ms or s, depending on the experiment) on the *x*-axis and the peak integral on the *y*-axis was fitted to the equation *y* = *B* + *F* · exp (–*x* · *G*). The T_1_ was then calculated as T_1=1/G_. The inversion recovery pulse sequence and data analysis scheme are reported in the Supporting Information (Figure S2: inversion recovery pulse sequence and data processing).

### 2.9 Wastewater Analysis

The wastewater sample was autoclaved and filtrated under vacuum on a 5–13 μm paper filter. Different amounts of polymeric material were weighed in 28 mL glass scintillation vials (10, 20, 40, 80, 160 mg) to test different solid-to-liquid ratios. Ten mL of sample were then added to the vials. Vials were then vigorously shaken at room temperature for 2 h. After this time, 1.7 mL were withdrawn and filtrated on a 0.22 μm filter syringe to perform the IC analyses. Binding experiments were performed in triplicate.

## 3 Results and Discussion

### 3.1 Material Characterization

**Figure 1:**
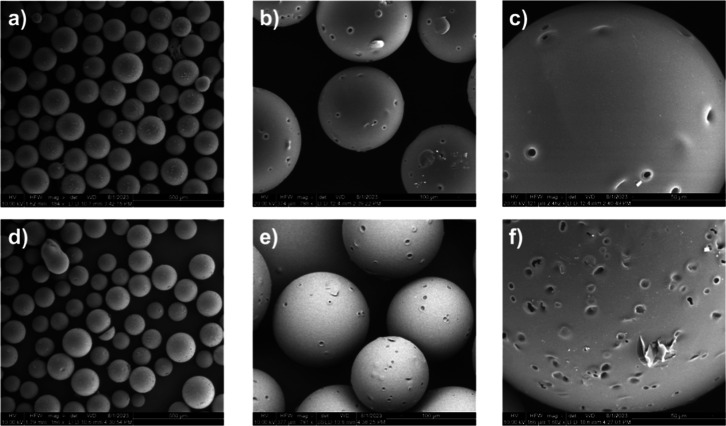
SEM imaging of the polymer VBC–HPMA–EGDMA before (a–c) and after (d–f) functionalization with TMA.

SEM images were obtained to confirm the spherical shape of the beads of the fraction of interest (120–250 μm; 17.5 g, 41% yield). The synthesis was considered successful as most of the material has a spherical shape, even though small aggregates and leftover PVA were present. After the reaction and the swelling with TMA, the shape and beads dimensions did not change substantially (Figure 1).

**Figure 2:**
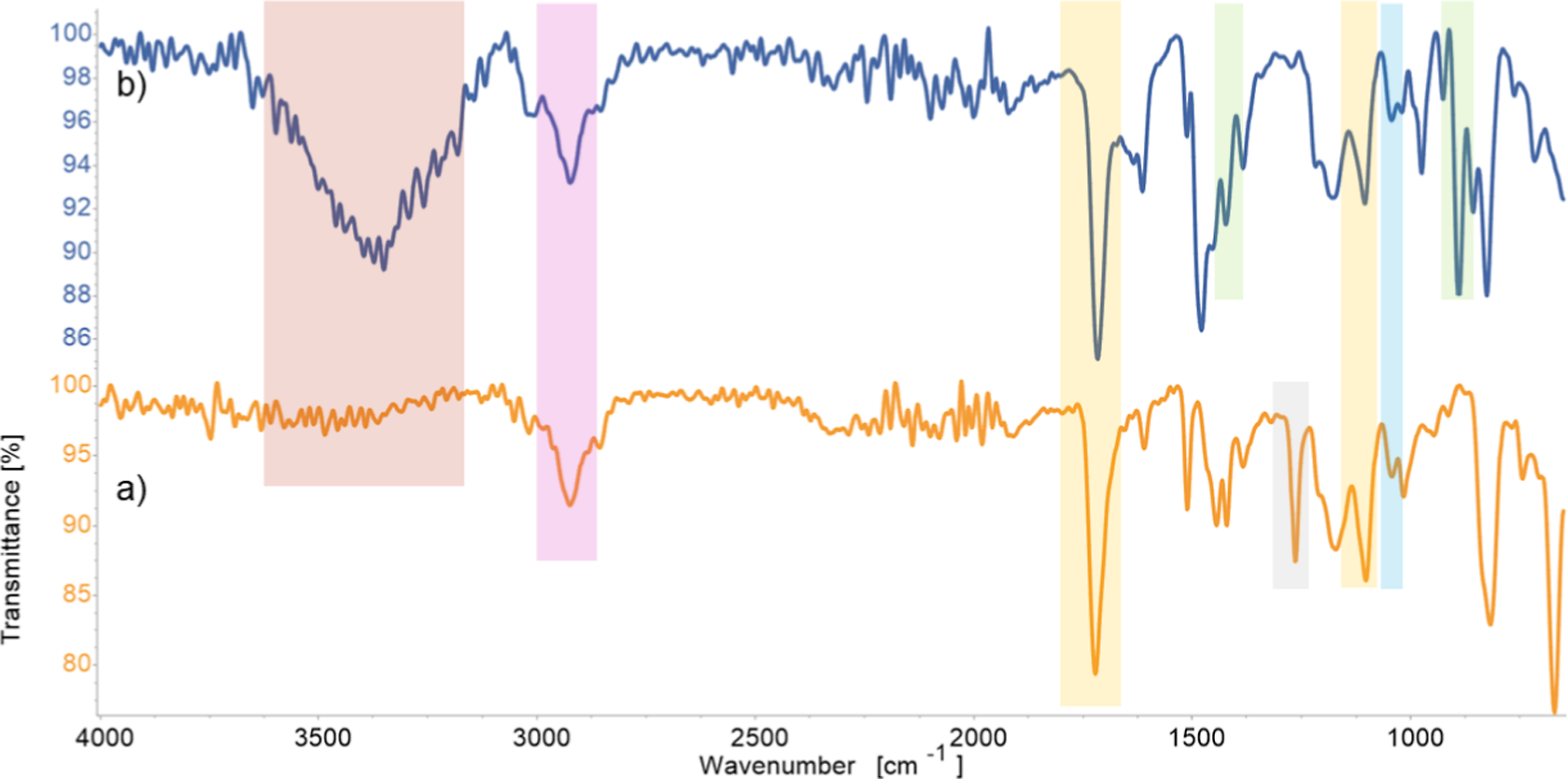
FTIR spectra of the polymeric beads before (a) and after (b) reaction with TMA. Bands of interest are highlighted in different colors. Green: N–CH_3_ stretching (890 cm^–1^) CH_3_ bending (1479 cm^–1^). Blue: C–O stretching (1103 cm^–1^). Yellow: C–O stretching (1176 cm^–1^), C=O stretching (1720 cm^–1^). gray: C–Cl stretching (1265 cm^–1^). Pink: C–H stretching (2924 cm^–1^). Red: O–H stretching (3350 cm^–1^).

The completion of the reaction with TMA, which produced a quaternary-ammonium functionalized polymer with 91% yield, was assessed using CNHS, FTIR, and solid-state NMR. Elemental analyses (Table S1: Elemental analysis of the polymeric materials) confirmed the addition of the quaternary ammonium group, evidenced by the increase in total nitrogen content. FTIR spectra (Figure 2) show the CH_2_–Cl stretch at 1265 cm^–1^, which disappears after the reaction with TMA, while a new band at 890 cm^–1^ appears, corresponding to the quaternary ammonium stretch. Furthermore, an additional bending vibration of the methyl group appears at 1479 cm^–1^ after the reaction with TMA. The C–H bends in the aromatic zone (800–690 cm^–1^) and the C=C stretching (1625–1440 cm^–1^) further confirm that the VBC benzyl group was incorporated in the polymeric material. In both spectra, the carbonyl stretching at 1720 cm^–1^ and the C–O stretching at 1176 cm^–1^ confirm the esters incorporation, while the secondary alcohol C–O stretching at 1103 cm^–1^ indicates the presence of the hydroxypropyl methacrylate monomer. In both spectra, the alkane C–H stretching at 2924 cm^–1^ indicates that all the monomers in the polymers have reacted, thus forming C–C bonds from the vinyl groups via free radical polymerization. The O–H stretching around 3350 cm^–1^ is present but weak in the material before the TMA reaction, while the signal is strong after the reaction with TMA. This is attributed to the presence of residual reaction solvent, as no evidence of monomer hydrolysis that would result in free alcohol groups was detected.

**Figure 3:**
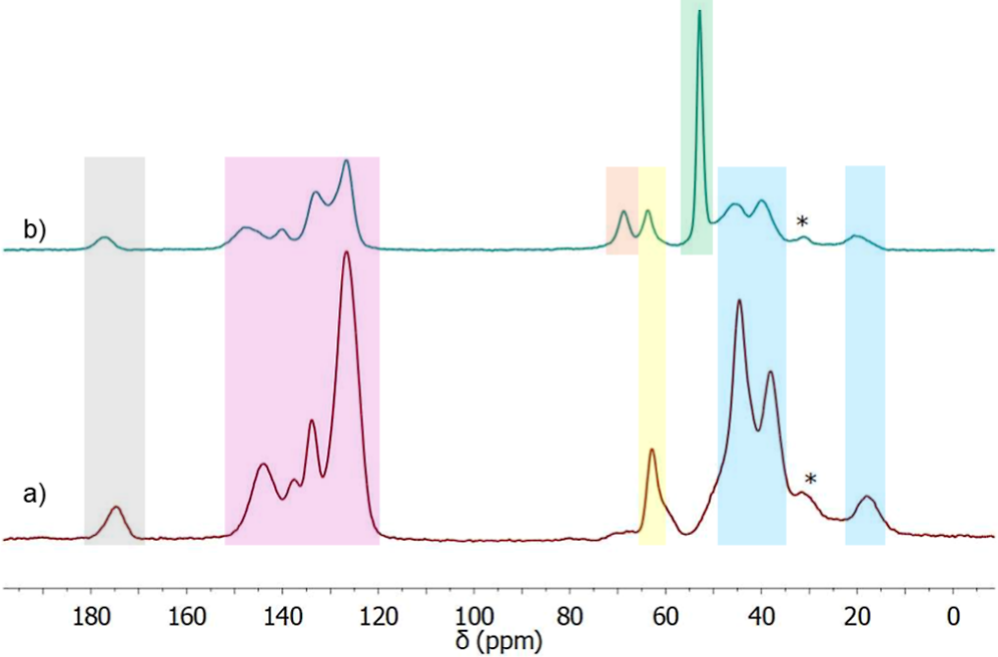
^13^C CP MAS spectra of the polymer VBC-HPMA-EGDMA before (a) and after (b) functionalization with TMA. Spinning sidebands are indicated by an *. Light blue: aliphatic signals (19, 40.4, and 45.8 ppm); green: N–^13^CH_3_ signal (53.3 ppm); yellow: ^13^C–O signal (62.9 ppm); orange: ^13^C–N signal (69.5 ppm); pink: aromatic signals (127–147 ppm); gray: ^13^C=O signal (177.7 ppm).

Figure 3 shows the ^13^C CP MAS spectra obtained before and after the TMA reaction. The peak at 19 ppm corresponds to the –CH_3_ groups of the monomer HPMA, which appears at 15.4, 17.4, and 18.7 ppm in the monomer ^13^C solution NMR spectra. The two aliphatic peaks at 40.4 and 45.8 ppm are attributed to the chain formed after the polymerization reaction: these are absent in all the monomer spectra, so they correspond to newly formed C–C bonds. The peak at 53.3 ppm in the blue spectra corresponds to the N–CH_3_ methyl groups as it appears after the functionalization reaction, and it confirms the formation of the quaternary ammonium group. A peak at 52.9 ppm in the solution NMR ^13^C spectrum of vinyl-benzyl trimethylammonium chloride supports this hypothesis. Before functionalization, the spectra show a broad peak at 62.9 ppm with a shoulder corresponding to most C–O and C–Cl carbons. After the functionalization reaction, the shoulder disappears, the peak slightly shifts to 64.2 ppm, and a new peak appears at 69.5 ppm. This new peak is attributed to the C–N group, which connects the aromatic ring to the quaternary ammonium group, while the peak at 64.2 ppm is the average of the C–O in the polymer structure. In the aromatic zone, there are four peaks attributed to the aromatic ring of the vinyl-benzyl moiety. This part of the spectrum changes the least before and after the reaction with TMA, as, during the reaction, an electronegative atom (Cl) switches with another electronegative atom (N). Finally, the peak at 177.7 ppm is attributed to the carbonyl groups of HPMA and EGDMA.

### 3.2 Isotherm and Kinetics

**Figure 4:**
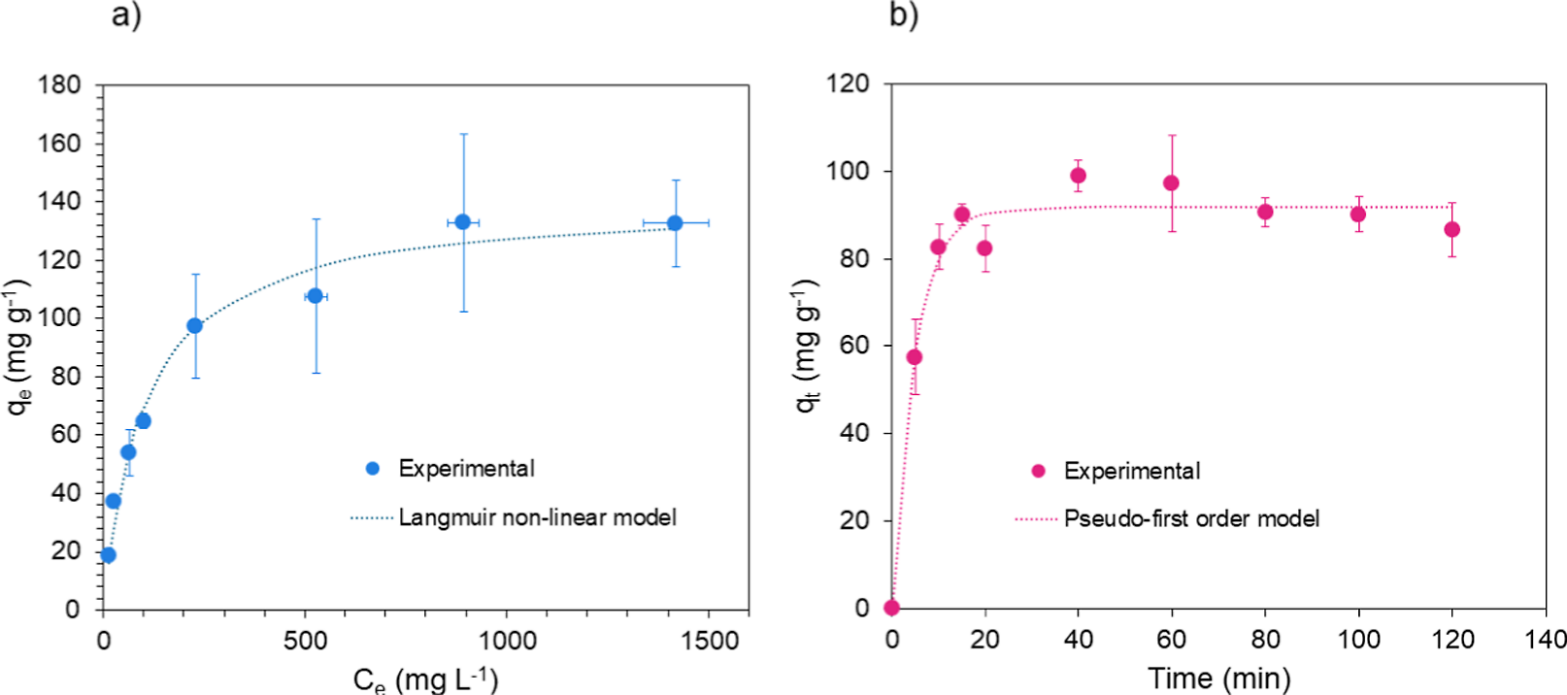
(a) binding isotherm of the polymer with H_2_PO_4_^–^. The isotherm is fitted with the Langmuir model (*R*^2^ = 0.981); (b) binding kinetics of the polymer with H_2_PO_4_^–^.

The binding isotherm obtained was fitted to the langmuir model, with a good correlation coefficient (*R*^2^ = 0.981) (Figure 4a). This can be explained as the electrostatic interactions involved between the sorbent and the adsorbate form a monolayer, with a homogeneous distribution of the binding sites.^32^ The calculated *q*_max_ for this material is 140.1 mg (H_2_PO_4_^–^) per g, while the Langmuir affinity constant *K* is 0.0098 L mg^–1^. The *K* value is attributed to the binding mechanism involved: generally, in the presence of physisorption, the affinity toward the adsorbate is not high, when a covalent bond is involved, H_2_PO_4_^–^ affinity constants can reach values up to 0.3 L mg^–1^.^33^

Adsorption kinetics experiments (Figure 4b) confirmed that the polymer’s saturation occurs within 15 min of contact with the adsorbate solution. The experimental values were fitted to the pseudo-first-order model, which had a better fit (*R*^2^ = 0.975) compared to the pseudo-second-order model (*R*^2^ = 0.959). As the pseudo-first-order model fits best when physisorption happens, this confirms that the binding mechanism is based on electrostatic interactions.^34^

### 3.3 Flow Experiments

**Figure 5:**
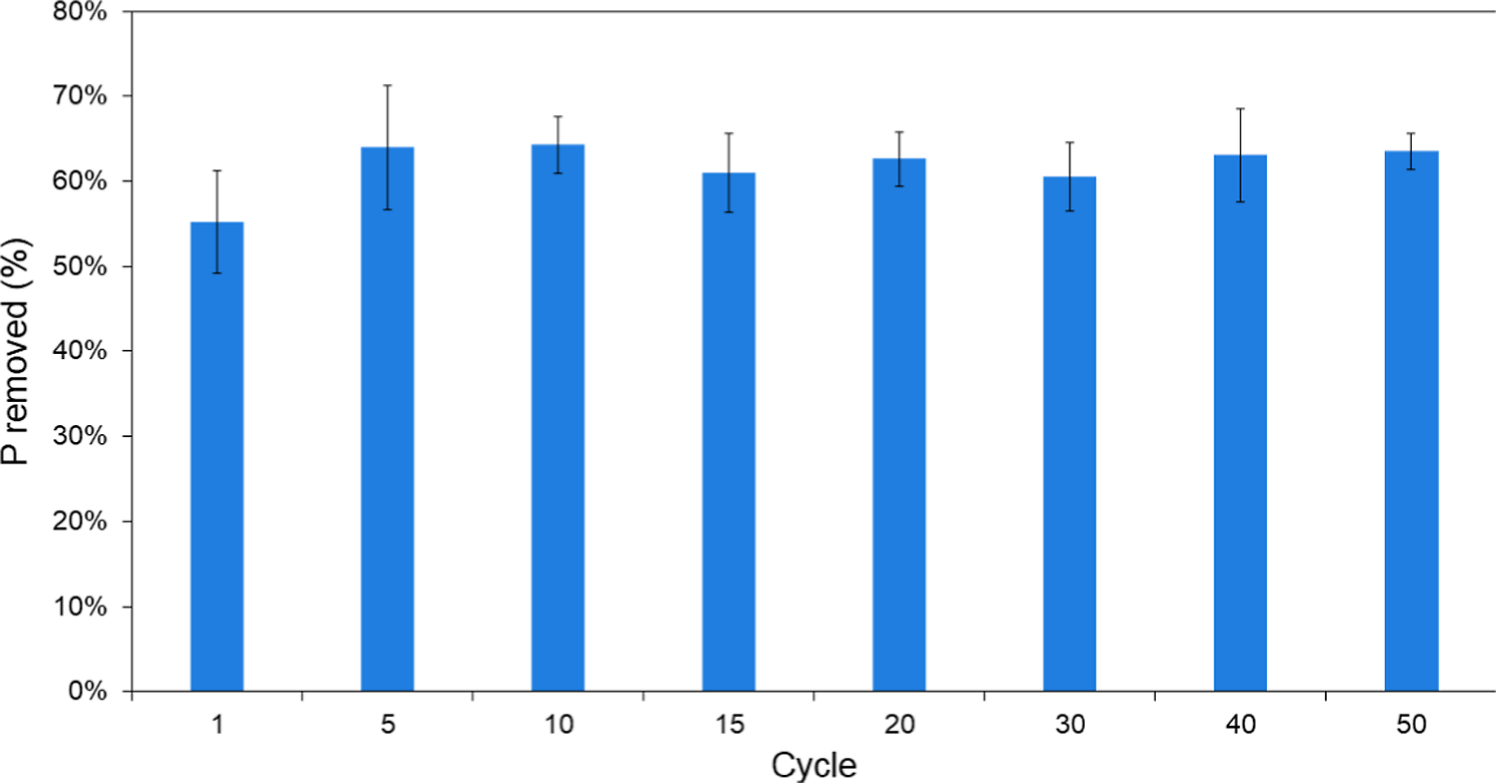
P removed from solution (%) at each cycle (*n* = 3).

Fixed-bed flow experiments confirmed that the adsorption capacity of the polymer did not change after 50 cycles of regeneration (Figure S3: Breakthrough curves at the different regeneration cycles). However, analysis of variance performed on the results of the regeneration study revealed a statistical difference in the first three sampling points (*p* < 0.05 for the 5, 10, and 15 min points). To investigate the source of variation, outlier analysis and Dunnett’s test were conducted. It was confirmed that the source of variation is given by the first binding cycle. It is hypothesized that this could be attributed to the time necessary for the gel-type polymer to swell in the aqueous medium. Once the polymer swells, the binding sites are more accessible for interaction with the adsorbate, and the binding becomes comparable for all subsequent regeneration cycles. Figure 5 shows the percentage of phosphate removed across various regeneration cycles. As expected from the breakthrough curves, the removal rate is the lowest during the first binding cycle, with only 55% of phosphate removed. However, after the fifth cycle, the removal rate stabilizes, ranging between 61% and 64% of phosphate removed.

### 3.4 Assessment of Polymer Stability

Polymer stability during repeated adsorption/regeneration cycles was assessed by solid-state NMR. Initially, ^31^P SP MAS experiments confirmed the complete recovery of phosphate upon desorption. Figure S4 shows the ^31^P SP MAS spectra of the polymer after binding phosphate and after the different regeneration cycles. Upon binding H_2_PO_4_^–^, the phosphorus signal appears as a single sharp peak at 0.77 ppm. Compared to the ^31^P SP spectra of monobasic potassium phosphate (KH_2_PO_4_, Figure S5: ^31^P SP spectrum of KH_2_PO_4_), this is shifted to a higher field due to dissociation in aqueous solution, followed by binding to the polymer. However, the sharp, symmetrical peak confirms that the binding mechanism is mainly due to physisorption (e.g., electrostatic interactions). Spectra obtained upon polymer regeneration do not show any ^31^P signal, confirming that the desorption solution successfully removes all P from the polymer.

**Figure 6:**
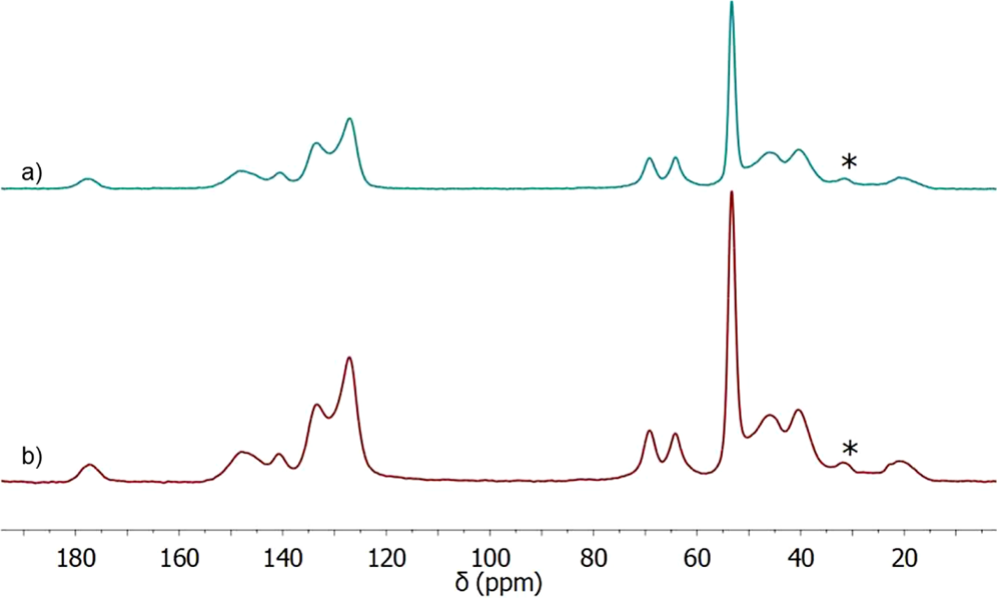
^13^C CP MAS spectra of the initial polymer (a) and after 50 cycles of regeneration (b). Spinning sidebands are indicated by an *.

The spectra of the polymer after synthesis, and after the 50th regeneration cycle, are shown in Figure 6. As shown, the spectra corresponding to the material after one and 50 regeneration cycles are essentially identical, suggesting the chemical composition of the polymer was not affected.

The spin–lattice relaxation times (T_1_) were measured for ^1^H and ^13^C nuclei to investigate the material’s stability further. The ^1^H T_1_ trend in the polymer after the different regeneration cycles is reported in Figure S6 and the T_1_ numerical values are reported in Table S2. It is found that within the first 30 cycles, the RSD % in T_1_ values is in the range of 2–3%, with T_1_ values around 550–590 ms. At the 50th cycle of regeneration, there is an increase in the values of 20–60 ms, depending on the peak. However, the overall RSD % of the T_1_ between 1 and 50 regeneration cycles is between 3% and 4%. The exceptionally consistent ^1^H T_1_ values suggest that the polymer is stable over 50 cycles of regeneration.

The ^13^C CP T_1_ values at the different regeneration cycles are similar, and the largest variation is for the peak at 177.7 ppm (C=O), with an RSD of 17% (Table S3: ^13^C T_1_ values for the polymer at different regeneration cycles). This variance might be due to variations in the sample preparation or a change in the chemical environment, more likely in the ester bonds of the monomer HPMA and EGDMA. Although the concomitant nitrogen content decrease found by elemental composition analysis could support the hypothesis of polymer degradation, the highest variation in the T_1_ values is between the 30th and 50th regeneration cycle. This could suggest an experimental error or that the polymer does not undergo any substantial degradation for at least 30 regeneration cycles. However, as no decrease in capacity was detected when increasing the regeneration cycles, the material was considered robust and reusable for at least 50 cycles.

### 3.5 Competitive Binding Assay and Wastewater Treatment

**Figure 7:**
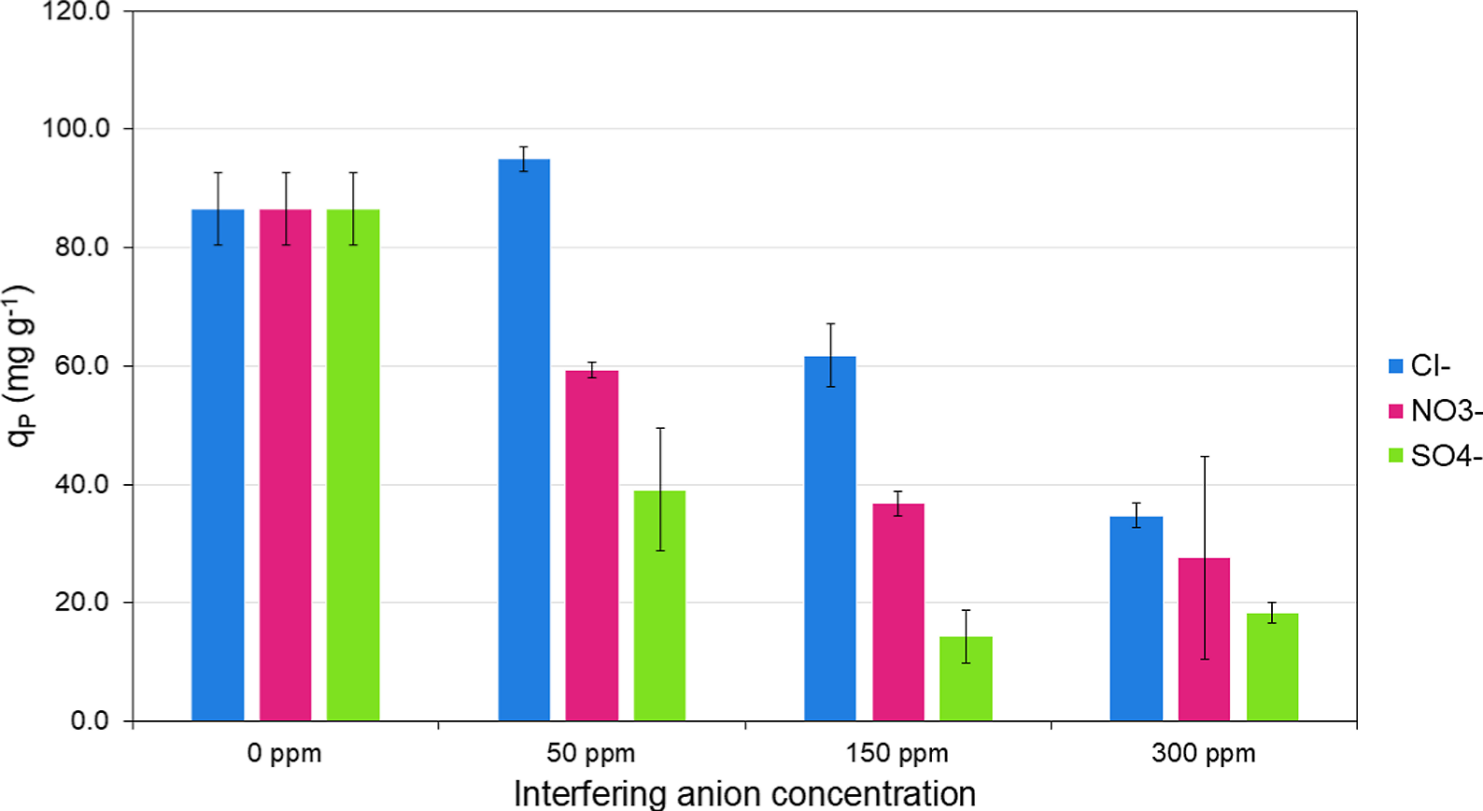
Binding capacity toward H_2_PO_4_^–^ in the presence of competing anions at different concentrations.

The binding competition assay with other anions, shown in Figure 7, suggests that most anions compete with phosphate for binding on the polymeric material. This was expected due to the main mechanism involved in the binding, based on electrostatic interactions. The main competitor for the binding is sulfate (SO_4_^2–^), due to its tetrahedral structure and high hydration, which are very similar to phosphate (H_2_PO_4_^–^). However, it was found that high concentrations of chloride and nitrate interfere with phosphate binding and reduce the polymer binding capacity by around 60%.

**Figure 8:**
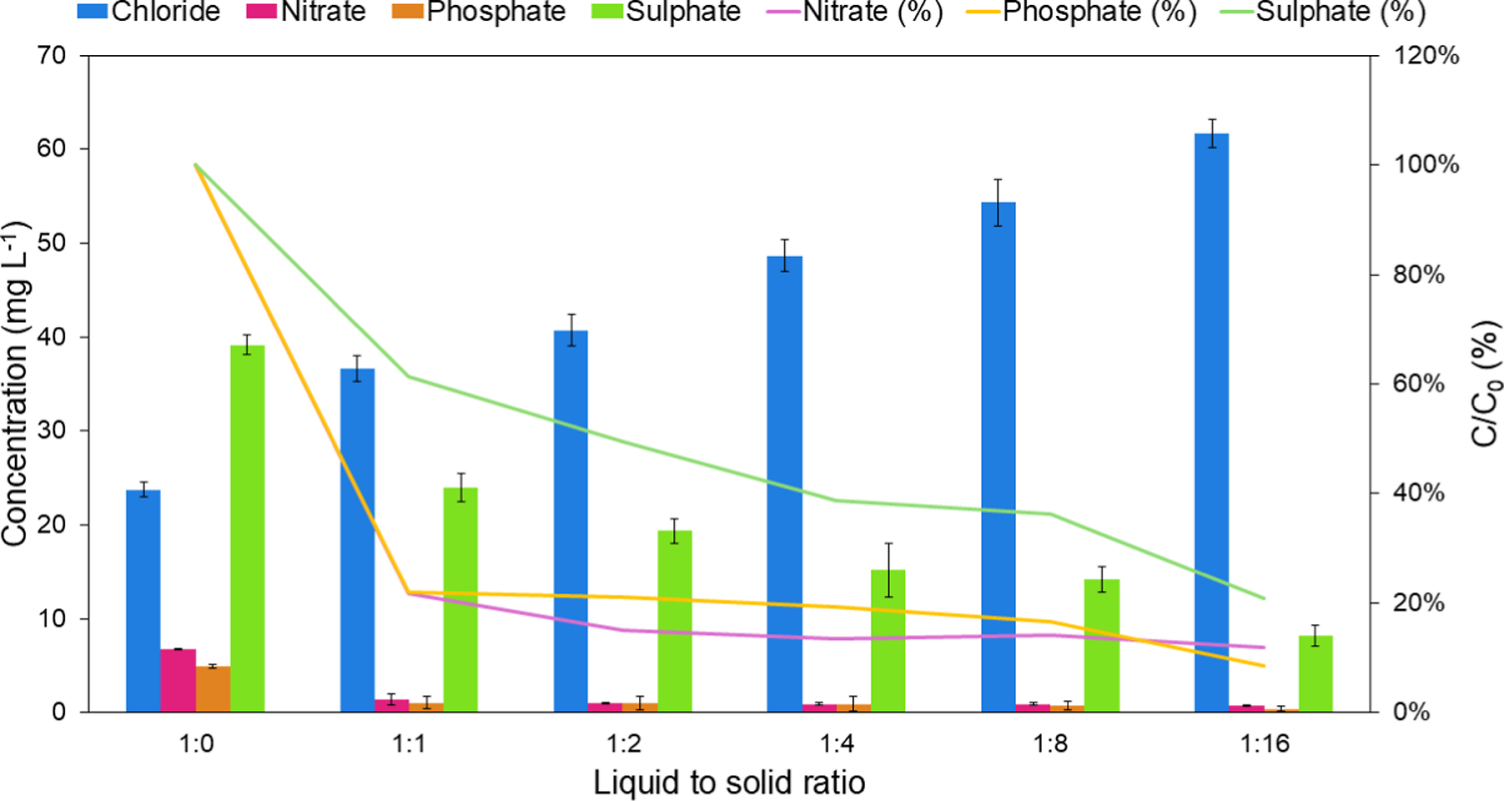
Anions concentrations in the wastewater sample at different liquid-to-solid ratios of the polymeric material tested.

Figure 8 shows the change in anion concentration in the wastewater sample when the amount of polymeric material is increased. It was shown that in the presence of competing anions at elevated concentrations, the polymer removes 78% of phosphate even when the lowest amount of polymer is used. The largest phosphate removal (91%) was observed with the lowest liquid-to-solid ratio tested, overcoming the interference observed by the other anions. It was also noted that after exposure to the polymeric material, chloride concentration in the samples increased due to the anion exchange process.

## 4 Conclusions

In this work, a polymeric sorbent for phosphate recovery was synthesized via suspension polymerization and characterized by SEM, elemental analysis, FTIR, and ssNMR. The synthesis produced uniform spherical beads that were functionalized with a quaternary ammonium group. Binding evaluation of the resulting sorbents revealed a maximum capacity of 137.2 mg g^–1^, outperforming commercially available and previously reported materials (Table 1), whereas fitting of the kinetic isotherms to the pseudo-first-order model confirmed that the binding mechanism is primarily electrostatic. Repeated adsorption/regeneration cycles revealed that sorbent binding capacity is not affected by regeneration with a strong acid solution providing valuable information about the stability of these materials. No significant variation was observed in the ^13^C CP MAS spectra, in the ^1^H SP spin–lattice relaxation times (T_1_), and in the ^13^C CP spin–lattice relaxation times (T_1_). Furthermore, the binding capacity was not affected during at least 50 adsorption/regeneration cycles. Upon application of the polymer in competitive binding of phosphate in the presence of other common anions in artificial samples, it was shown that sulfate was the main competitor for the binding sites. However, upon testing with domestic wastewater samples, the polymer demonstrated a high affinity toward phosphate. The high phosphate binding capacity, coupled with the ability to recover bound phosphate in a directly useable form, and long-term adsorption/desorption stability, make the materials presented here strong candidates for application as phosphate binding sorbents in real-world applications, such as the recovery of phosphate from industrial effluent streams, wastewater, or freshwater systems.

**Table 1:** Comparison Among the Langmuir Calculated *q*_max_ and Regeneration Cycles Studied for Materials Reported in Literature and the Polymer Reported in This Work

material type	regeneration cycles	*q*_max_ (mg PO_4_^3–^/g)	references
La_2_(CO_3_)_3–_ loaded strong anion exchange resin	5	52.9 (25 °C)	(35)
porous Mg-based cementitious material	not tested	42.2 (25 °C)	(36)
Fe/Mn loaded strong anion exchange resin	not tested	51.0 (30 °C)	(37)
gel-type strong anion exchange resin	not tested	38.0 (30 °C)	(37)
hybrid ion-exchange resin (Layne^RT^)	63	39.0 (25 °C)	(30)
PVA/sodium alginate double network hydrogels loaded with La(III)	8	38.7 (25 °C)	(38)
gel-type anion exchange polymer	50	137.2 (25 °C)	this work

## Supporting Information Available

Material synthesis scheme, additional solid-state NMR spectra and calculated parameters (PDF)
