# Supplementary material for: Evaluation of Performance and Stability of a Gel-Type Polymer Sorbent for Recovery of Phosphate from Waste Streams
Source: ACS Appl Polym Mater. 2024 Dec 6;6(24):15304–12. doi: 10.1021/acsapm.4c03237 (PMC11686460; doi:10.1021/acsapm.4c03237)
Supplement: Supplementary file 1 — ap4c03237_si_001.pdf [file ap4c03237_si_001.pdf]

Supporting Information

Evaluation of performance and stability of a gel-type polymer sorbent for recovery of phosphate from waste streams

Michela Pacchione [a], *Lucas Urbano José* [b], *Ulla Gro Nielsen* [b], *John W. McGrath* [c], *Panagiotis Manesiotis* [a,*]

[a] School of Chemistry and Chemical Engineering, Queen's University, Belfast, David Keir Building, Stranmillis Road, BT9 5AG, Belfast, Northern Ireland, UK

[b] Department of Physics, Chemistry and Pharmacy, University of Southern Denmark, Campusvej 55, Odense, DK

[c] School of Biological Sciences, Queen's University, Belfast, 19 Chlorine Gardens, BT9 5DL, Belfast, Northern Ireland, UK

*Corresponding author: p.manesiotis@qub.ac.uk

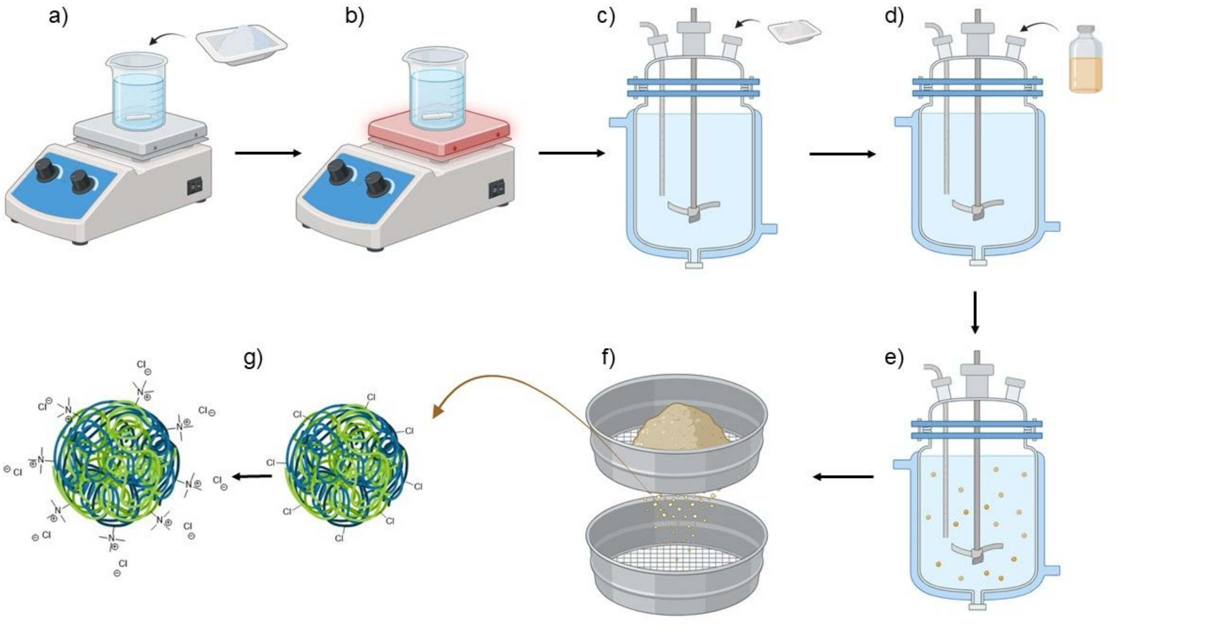

Figure S1: Synthesis scheme for the polymeric beads. a) Preparation of the PVA solution by dissolving 9 g of PVA in 837 mL of ultra-pure water; b) heating at 90 ˚ C under magnetic stirring overnight to completely dissolve the PVA; c) transfer of the PVA solution in the reaction vessel and addition of 54 g of NaCl, while stirring at 250 rpm at 70 ˚ C; d) addition of the oil phase under stirring at 250 rpm at 70 ˚ C; e) free-polymerization reaction proceeds for 8h under stirring at 250 rpm, 70 ˚ C; f) beads collected from the reactor are washed and sieved; g) beads of the fraction of interest (120-250 μm) are functionalized with TMA at 60 °C in ethanol.

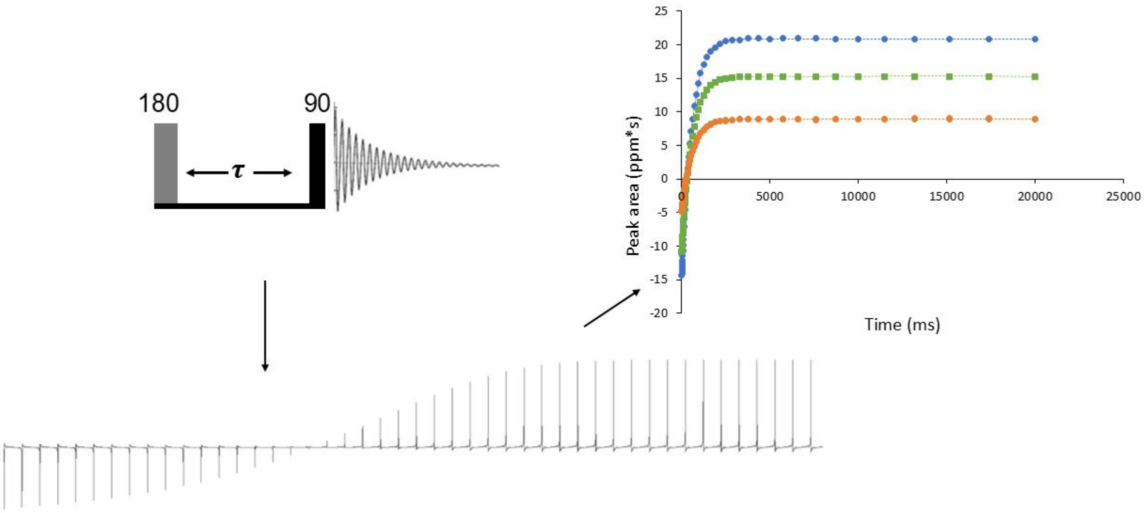

Figure S2: ^{1}H inversion recovery pulse sequence and data processing scheme. The experiment is performed by applying a 180° pulse on the x axis, followed by a time delay (τ). Then, a 90° pulse is applied, and the FID is collected. The resulting plot is a 2D plot (integral *vs* time) that can be fitted with the equation $y = B + F \cdot exp\,(-x \cdot G)$. τ (Equation S1) varies between 30 and 2000 ms for the ^{1}H inversion recovery (48 points) and between 0.1 and 10 s for the ^{13}C CP inversion recovery (48 points).

Table S1: Calculated and experimental composition for the polymer before the functionalization with TMA, after the functionalization with TMA, after 30 cycles of regeneration, and after 50 cycles of regeneration.

	Before TMA functionalization		After TMA functionalization			
**Element (%)**	**Calculated**	**Experimental**	**Calculated**	**Experimental**	**After 30 cycles**	**After 50 cycles**
**C**	68.60	70.21	66.78	58.77	60.4	60.13
**H**	6.35	6.17	8.49	8.22	8.97	8.99
**N**	0.29	<0.3	5.87	3.47	2.25	2.12
**S**	<0.3	0.65	<0.3	0.35	<0.3	<0.3

The polymer's elemental composition is slightly different from what is expected after the functionalization with TMA. This might be because not all the benzyl-chloride groups are accessible for the functionalization with the amine. However, after repeated regenerations, the nitrogen content slightly decreases, while the carbon and hydrogen content increases.

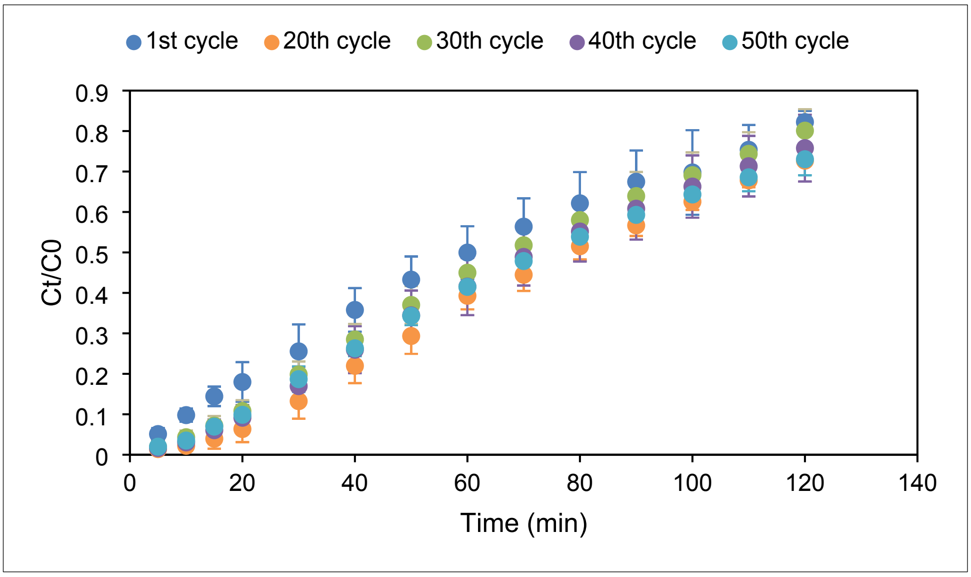

Figure S3: Breakthrough curves at the different regeneration cycle for the binding of 100 ppm $H_2PO_4^-$ on the polymer.

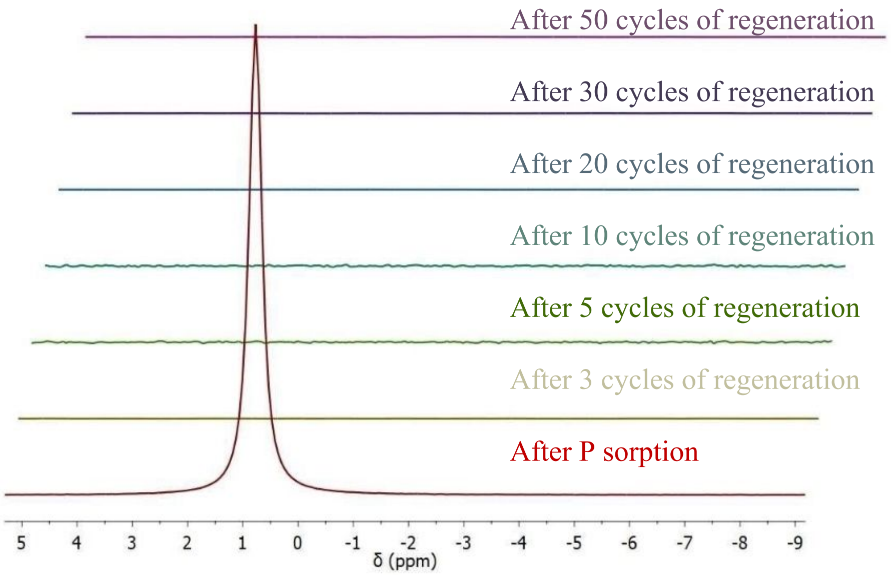

Figure S4: ^{31}P SP MAS spectra of the polymer VBC-HPMA-EGDMA-TMA after the binding with $H_2PO_4^-$ and after the different regeneration cycles.

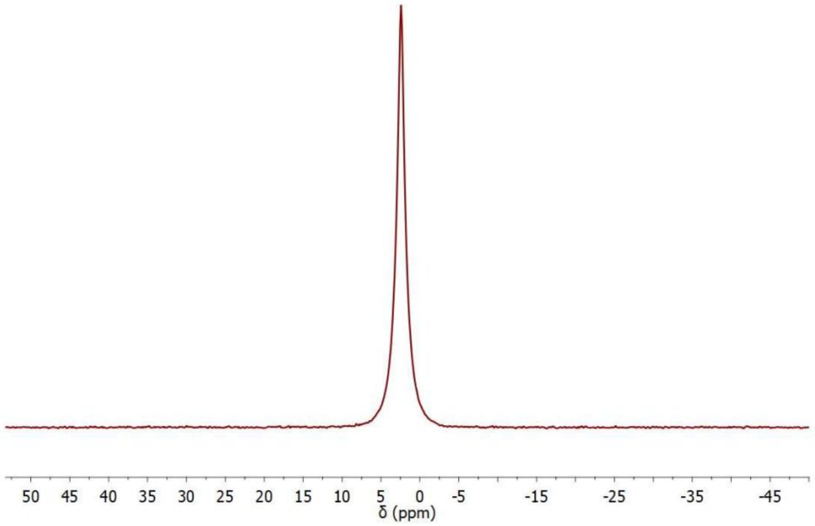

Figure S5: ^{31}P SP spectrum of KH_2PO_4. The spectrum shows as a single peak at 2.42 ppm.

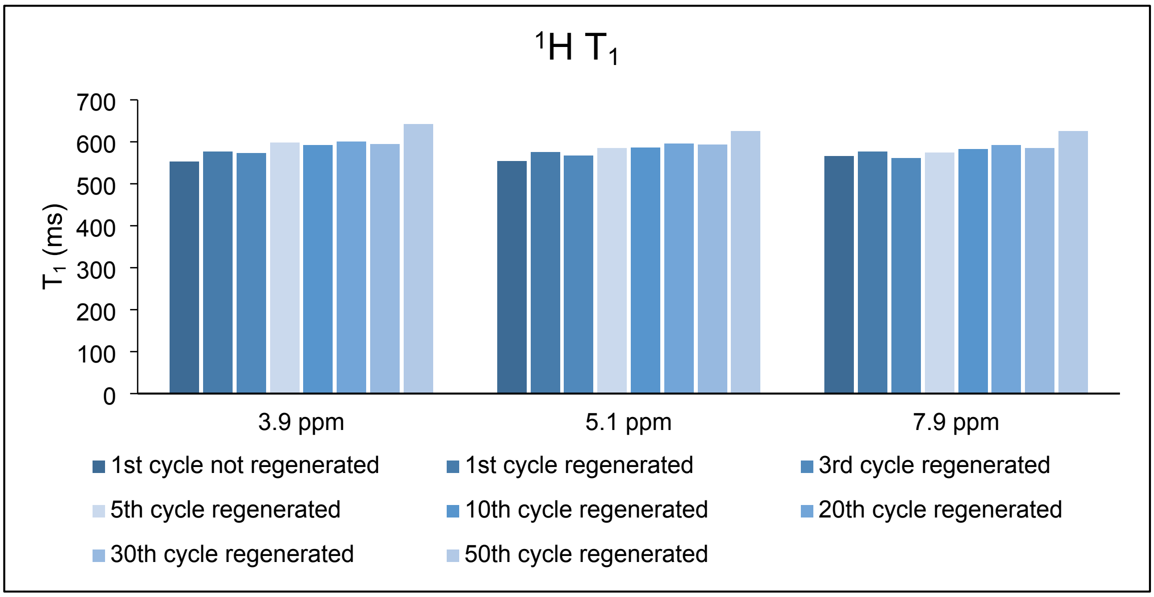

Figure S6: ^1H Spin-lattice relaxation time (T_1) calculated *via* inversion recovery experiment for the main peaks in the ^1H spectra of the polymer VBC-HPMA-EGDMA-TMA.

Table S2: ^1H T_1 values for the polymer VBC-HPMA-EGDMA-TMA at the different regeneration cycles.

**δ_{iso} (ppm)**	**3.9**	**5.1**	**7.9**
Cycle	T_1 (ms)		
0	552.5	554.3	566.4
1	576.0	574.8	575.9
3	572.6	566.5	561.2
5	597.8	585.3	573.8
10	592.2	586.6	582.1
20	600.9	596.1	592.0
30	594.4	593.0	585.4
50	641.7	624.9	625.5
**SD (0-30th cycle)**	16.2	13.9	9.9
**Average**	583.8	579.5	576.7
**RSD**	3%	2%	2%
**SD (all cycles)**	24.4	19.9	18.6
**Average**	591.0	585.2	582.8
**RSD**	4%	3%	3%

Table S3: ^{13}C T_1 values for the polymer VBC-HPMA-EGDMA-TMA at the different regeneration cycles.

**δ_{iso} (ppm)**	**40.4**	**45.8**	**53.3**	**64.2**	**69.5**	**127.3**	**138.3**	**177.7**
Cycle	T_1 (s)							
0	0.565	0.558	0.514	0.531	0.583	0.579	0.583	0.625
1	0.543	0.583	0.537	0.543	0.753	0.567	0.589	0.631
3	0.555	0.593	0.618	0.593	0.503	0.621	0.593	0.596
5	0.615	0.578	0.573	0.641	0.611	0.571	0.573	0.528
10	0.583	0.594	0.580	0.627	0.609	0.596	0.583	0.513
20	0.593	0.599	0.607	0.580	0.724	0.603	0.606	0.549
30	0.554	0.611	0.609	0.624	0.687	0.607	0.609	0.673
50	0.664	0.649	0.651	0.721	0.578	0.651	0.662	0.860
**SD**	0.037	0.025	0.042	0.056	0.078	0.026	0.026	0.103
**Average**	0.584	0.595	0.586	0.607	0.631	0.599	0.599	0.621
**RSD**	6%	4%	7%	9%	12%	4%	4%	17%
